# Development, education, and services in children with Down syndrome: a cohort analysis from a clinical database

**DOI:** 10.3389/fpsyg.2024.1348404

**Published:** 2024-10-24

**Authors:** Cara Soccorso, Margaret Hojlo, Katherine Pawlowski, Angela Lombardo, Emily Davidson, Sabrina Sargado, Rafael DePillis, Nicole Baumer

**Affiliations:** ^1^Division of Developmental Medicine, Boston Children’s Hospital, Boston, MA, United States; ^2^Harvard Medical School, Boston, MA, United States; ^3^Department of Neurology, Boston Children’s Hospital, Harvard Medical School, Boston, MA, United States

**Keywords:** Down syndrome, development, developmental milestones, behavior, education, services, interventions

## Abstract

**Background:**

Prior research has characterized neurodevelopmental phenotypes for Down syndrome (DS), but there is variability in age of milestone attainment and limited identification of early predictors of developmental trajectories. Additionally, less is known about receipt of education and services in relation to development.

**Objective:**

This study describes the delivery of education and therapies in the setting of general developmental and behavioral needs in a large clinical cohort of children with DS seen in a specialized Down Syndrome Program (DSP).

**Method:**

The clinically collected data included 814 patients with DS who were seen at a specialty DSP at a large, tertiary pediatric care center from March 2018 to January 2023. Data were collected through caregiver-and clinician-reported history at clinical visits to the program. Descriptive frequencies were utilized to describe participant demographics, skills and behaviors, and receipt of services, across age groups in childhood.

**Results:**

Delays were present across all developmental domains; in particular delays in language, communication, and academic skills, and behavioral challenges were commonly reported. Almost all children received Early Intervention (EI) services, and many young children received non-public therapies after completing EI. Older participants demonstrated more impairments than younger age groups, yet received services at lower rates, particularly behavioral and speech language interventions.

**Conclusion:**

A snapshot of developmental skill attainment in individuals with DS is provided. Therapies to support the levels of need were reported at much lower frequencies than the level of need reported to target aspects of development and behavior. Several gaps in therapies and educational services were identified. There is an important need for tailoring supports, based on developmental level, to meet individual needs. These findings may help to inform policy change related to developmental and educational services for individuals with DS.

## Introduction

Down syndrome (DS), caused by all or part of an extra chromosome 21, is the most common chromosomal condition (Roizen and Patterson, 2003). Prior research has characterized a neurocognitive phenotype for DS in which cognitive, language, behavior, social, and adaptive skills vary greatly (Chapman and Hesketh, 2001; Martin et al., 2009; Dykens et al., 2006; Onnivello et al., 2023; Frank and Esbensen, 2015). For instance, children with DS have strong visual-matching abilities and are capable of learning sight words at a very young age (between 2.5 and 3.5 years) (Buckley and Bird, 1993). Steady establishment of adaptive skills (i.e., daily living activities) is also seen in young children with DS, though these skills may plateau in later years (Dykens et al., 2006). Sociability, including interest in social engagement and play, is regarded as a relative strength for many individuals of all ages with DS (Sigman and Ruskin, 1999), while communication remains an area of relative weakness, with expressive skills even less developed than receptive skills (Dykens et al., 2006).

Though broader studies on childhood development often do not include children with DS (Hendrix et al., 2021), it has been estimated that, on average, children with DS reach developmental milestones 1.5–2 times later than typically developing children (Vellody, 2020). The variability in age of milestone attainment and limited identification of early predictors of developmental trajectories have resulted in challenges with identifying individuals with DS with atypical development, projecting developmental outcomes, and planning individualized interventions (Luyster et al., 2011).

Little is known about the nature of educational and therapeutic interventions received by children with DS in the United States (U.S.). Because infants and toddlers up to age three with DS can be considered “at-risk” under the U.S. federal statute establishing Early Intervention (EI) services (Individuals with Disabilities Education Act, 1975), all children under three years of age with a DS diagnosis should qualify for government-funded EI services to support development (e.g., speech and language therapy, occupational therapy); however, it is not known how often these services are accessed. Additionally, due to different interpretations of this statute, there may be variable access to EI services by state (Barger et al., 2019). School placement and programming for individuals with DS are also variable (Hargreaves et al., 2021). New England, and specifically Massachusetts, is generally a well-resourced area with a high density and high accessibility to educational services (Friedman-Krauss and Barnett, 2023). There has been great advocacy over the years toward greater educational inclusion, including research which shows that children with DS who have inclusive school experiences (i.e., participating in classes alongside their typically-developing peers) have higher academic attainment compared to those with substantially separate programming (de Graaf et al., 2012).

While there have been increasing efforts in DS research in recent years, still there remain unmet needs including studies exploring developmental skills, behaviors, and service provision in DS (Hendrix et al., 2021). Here, we aim to describe: (1) developmental skills and behaviors in children with DS, and (2) the concurrent delivery of education and therapies, in a single large clinical cohort of children and adolescents with DS.

## Methods

### Participants

Children, adolescents, and young adults with DS (confirmed by clinical examination and/or karyotyping) who received care at a Down Syndrome Program (DSP) in a tertiary children’s hospital from March 2018 to January 2023, and who had completed at least one caregiver form about general development and/or education and services, were included (*N* = 814). As part of standardized clinical care, prior to each clinical visit, caregivers provided information regarding patients’ development, behavior, education, and services received. The data described below were collected prior to, during, and after the COVID-19 pandemic period, and data that were collected during the social isolation period were not excluded or separately analyzed. There were no changes to data forms collected during that time period.

### Measures

#### Sociodemographic

Zip code, date of birth, sex, race, and primary language were all caregiver-reported demographic fields collected at the time of the individual’s registration into the EMR.

#### Development

A “General Development” form, which was developed by the DSP team (Baumer et al., 2022), was given to children of all ages. For those older than age three years, a standardized caregiver-completed developmental monitoring tool, the Neurodevelopmental Parent Report for Outcome Monitoring (formerly the Autism Spectrum Disorder Parent Report for Outcome Monitoring; ASD-PROM) was also given. In its entirety, the ND-PROM consists of 127 Likert-scale style questions that assess general development and maladaptive behaviors, including communication, social skills, behavioral functioning, and adaptive skills (Levin et al., 2021). Seventeen of these questions, relevant to development, were selected for inclusion in this project (Table 1). Caregivers indicated “never,” “rarely,” “sometimes,” “often,” or “always” to each question. Skills were determined as “established” when caregivers responded that an individual was “often” or “always” doing the skill. Though initially used in autism spectrum disorder (ASD), the ND-PROM covers domains relevant for individuals with other developmental disabilities and neurodevelopmental conditions (Baumer et al., 2024).

**Table 1:** Source and use of variables.

Analysis area	Specific domain/category	Variables used	Variable source (instrument name)
Development	Language Skills (Figure 1A)	Follows 1-Step Directions	General Development
		Uses >50 Words/Signs/Phrases	General Development
		Uses 3–4 Word Sentences	General Development
	Social Communication Skills (Figure 1B)	Simple Pretend Play	General Development
		Points To Request^#^	ND-PROM
		Points To Show Interest	ND-PROM
		Simple Social Games	ND-PROM
		Social Interest*	ND-PROM
		Imitates Others	ND-PROM
	Communication Modality (Figure 1C)	Does Not Communicate	General Development
		Picture Communication System	General Development
		Electronic Communication Device	General Development
		Sign Language	General Development
		Spoken Language	General Development
	Gross Motor Skills (Figure 1D)	Runs	General Development
		Uses Stairs Independently	General Development
		Walks Independently**	General Development
	Adaptive Skills (Figure 1E)	Brushes Teeth	General Development
		Drinks From an Open Cup	General Development
		Toilet Trained	ND-PROM
		Dresses Self	General Development
	Academic Skills (Figure 1F)	Knows Basic Subtraction	General Development
		Knows Basic Addition	General Development
		Counts At Least 5 Objects	General Development
		Reads At Least 10 Words	General Development
		Writes Name	General Development
Behavioral Concerns	Behaviors (Figure 2)	Repetitive Movements	ND-PROM
		Repetitive Activities	ND-PROM
		Difficulties with Transition	ND-PROM
		Aggression Toward Self	ND-PROM
		Aggression Toward Others	ND-PROM
		Runs Away (Bolts or Wanders)	ND-PROM
		Caregiver’s Chief Clinical Concerns***	General Development
Education and Services	Receipt of Therapies (Figure 3A)	Behavioral Therapy↑	Education & Services
		Occupational Therapy (OT)	Education & Services
		Physical Therapy (PT)	Education & Services
		Speech Therapy	Education & Services
	Educational Placement (Figure 3B)	Inclusion	Education & Services
		Partial Inclusion	Education & Services
		Substantially Separate	Education & Services
	Family Services & Recreation Activities	Family Services^†^	Education & Services
		Recreational Activities^	Education & Services
	Barriers to Services (Table 3)	Waitlists	Education & Services
		Insurance Coverage/Finances	Education & Services
		Provider Availability	Education & Services
		Transportation	Education & Services
		Lack of Necessary Information About How to Access Services	Education & Services
		Program Not Willing/Able to Accommodate Child’s Needs	Education & Services
		Other	Education & Services

The General Development and Education & Services Instruments were administered to patients of all ages. The ND-PROM Instrument was administered to patients ages 3 years and older. ^#^ For the 0 < 3 years age group, responses to “Points to Indicate Need for Help” from the General Development form were used. * Item as written in the General Development form was “Seems Interested in Interacting with Peers.” ** Item as written in the General Development form was “Walks Around Home.” *** This is a qualitative variable derived from caregiver-reported Chief Clinical Concern at visit. ↑ Includes receipt of Applied Behavior Analysis and other behavioral therapies. † Includes engagement with: social work consultation, case management, counseling, childcare, personal care attendant, legal or financial services, educational advocate, mentorship, face-to-face or virtual peer support. ^ Includes engagement with: physical activity/exercise (e.g., non-therapy sports teams), social activities (e.g., spending time with friends), and religious activities (e.g., attending services). The General Development, Caregiver Chief Clinical Concerns, and Education & Services instruments were developed by the Down Syndrome Program team. More information about the creation and implementation of these instruments can be found in Baumer et al. (2022). More information about the creation and validation of the ND-PROM can be found in Levin et al. (2021). ND-PROM: Neurodevelopmental Parent Report for Outcome Monitoring.

#### Behavior

Information about behavior was derived from two sources for children ages three and older. First, the presence of behavioral concerns was defined as a report of “often” or “always” to the following items on the ND-PROM: Repetitive Movements; Repetitive Activities; Difficulties with Transition; Aggression Toward Self; Aggression Toward Others; and Running Away (e.g., bolting or wandering). Second, a caregiver form created by the DSP team (Baumer et al., 2022) that was presented to all patient families at the time of their visit to the DSP asked caregivers to report their most salient concerns at the visit (“Caregiver’s Chief Clinical Concerns”). The caregiver-reported behavioral information includes concerns at the time of a clinic visit and captures specific behavioral concerns, whereas the ND-PROM captures ongoing frequency of specific maladaptive behaviors (not specifically caregiver concerns), which was binarily coded as behavioral concern or no behavioral concern.

#### Education and services

Families were asked to complete an “Education and Services” form, which was also developed by the DSP team (Baumer et al., 2022) to share information about service receipt and activity participation across several domains: receipt of developmental and behavioral services (i.e., EI; as well as Behavioral Therapy, Occupational Therapy, Physical Therapy, and Speech Therapy received outside of EI or school); educational classroom placement (i.e., Inclusion setting, which is when a child is integrated into the general education classroom; Partial Inclusion setting, which is when a child is integrated into the general education classroom for some of the school day; and Substantially Separate setting, which is when a child is in a low teacher-to-student ratio environment rather than the general education classroom); Recreational Activities, which included physical activity/exercise (e.g., non-therapy sports teams), social activities (e.g., spending time with friends), religious activities (e.g., attending services), and non-school group activities; Family Services, which included supports such as case management, counseling, legal/financial services, and educational advocates; and barriers to services at the time of their clinical visits. Possible barriers included Insurance Coverage/Finances, Provider Availability, Transportation, and Lack of Necessary Information About How to Access Services.

### Procedures

Information was abstracted from the Electronic Medical Record (EMR) system and the DSP database for four key categories: (1) sociodemographic, (2) general development, (3) behavioral concerns, and (4) education and services. For a detailed summary of the data collection domains/categories, instruments, and variables used, please see the Table 1. Data were stored and managed using the REDCap (Research Electronic Data Capture) tool hosted at Boston Children’s Hospital (BCH), a secure, web-based software platform (Harris et al., 2009; Harris et al., 2019). This study was approved by the hospital’s Institutional Review Board.

*A priori* age categories (based on the age of the individual at the time of data collection) were established based on clinical determination of typical transitions in service delivery and development (0 to <3 years, 3 years to <5 years, 5 years to <7 years, 7 to <10 years, 10 to <14 years, and 14 to <18 years). Individuals who had data for more than one clinical visit were included in each of the age groups for which they had a visit. If multiple visits occurred within the same age-band, the most recent visit was used.

All children received initial forms with questions about developmental milestones at their clinical visits, in the areas of Language, Social Communication, Gross Motor, Adaptive, Academic skills, Education and Services; those over age three also reported on Behavior. Demographic data were collected from the EMR. Families with a primary language other than English completed all forms with the help of a hospital medical interpreter. Details of the variables and how they were derived are provided for clarity in Table 1. A full description of all clinically collected data, methodology, and data collection forms was included in a methodology-specific paper (Baumer et al., 2022).

### Analysis plan

The focus of the paper is to describe a large clinical sample; as such, statistical analysis of correlation was not undertaken. Samples of outlying data (e.g., children reported as reading before age two) were cross-checked with clinician reports in the EMR to reduce risk of misreporting and were corrected when appropriate. All unanswered variables, or variables marked as “not applicable,” were not counted in the calculation of the data percentage for a given variable. Data were summarized using descriptive statistics including age-based frequencies, percentages, and means, conducted using R 4.3.3 (R Core Team, 2024). Despite sex prevalence differences in the diagnosis of ASD, sex differences were not analyzed because there are no skewed sex prevalences of other co-occurring diagnoses (e.g., ADHD).

## Results

### Sociodemographics

The sociodemographics of 814 unique individuals included in the database from March 2018 through January 2023 are presented in Table 2. Ages ranged from 4 months to 18 years, with a median age of seven years. In this study, individuals were Asian (3%), Black/African American (7%), Multiracial (<1%), Native Hawaiian/Pacific Islander (<1%), White (64%), and Other (12%) (see Table 2). Patients in the database represented 87% percent of the total BCH DSP patient population who had clinical encounters during this time period (*N* = 940); the other patients did not return requested information in advance of their clinical visits.

**Table 2:** Population demographics and characteristics of individuals.

Population demographics and characteristics	*N* (%)
	*N* = 814
**Sex**	
Male	468 (57.5%)
Female	346 (42.5%)
Other	0 (0%)
**Age & database retention**	**(years)**
Median age	6.98 (IQR: 9.44)
Median time followed in the database	4.90 (IQR: 2.13)
	**(# of visits)**
Median number of visits captured in the database	4
**Race**	
White	522 (64%)
Black/African American	60 (7%)
Asian	20 (3%)
Native Alaskan/American Indian	0 (0%)
Native Hawaiian/Pacific Islander	2 (<1%)
Multiracial	2 (<1%)
Other	96 (12%)
Missing/Not reported	112 (14%)
**Median income based on Zip Code**	
≤$34,282	46 (6%)
≥34,282 but <54,850	314 (39%)
≥54,850 but <82,276	346 (43%)
≥82,276	103 (13%)
Missing/Not reported	5 (<1%)
**Primary language**	
English	722 (89%)
Spanish	43 (5%)
Portuguese	14 (2%)
Other	25 (3%)
Missing/Not reported	10 (1%)

For the purposes of the demographics table, median age represents age at first entry into database. Time followed in the database was calculated as the amount of time between first entry into database and April 12th, 2024. Income quartiles were estimated using median household income per patient zip code.

### Developmental patterns by age

**Figure 1:**
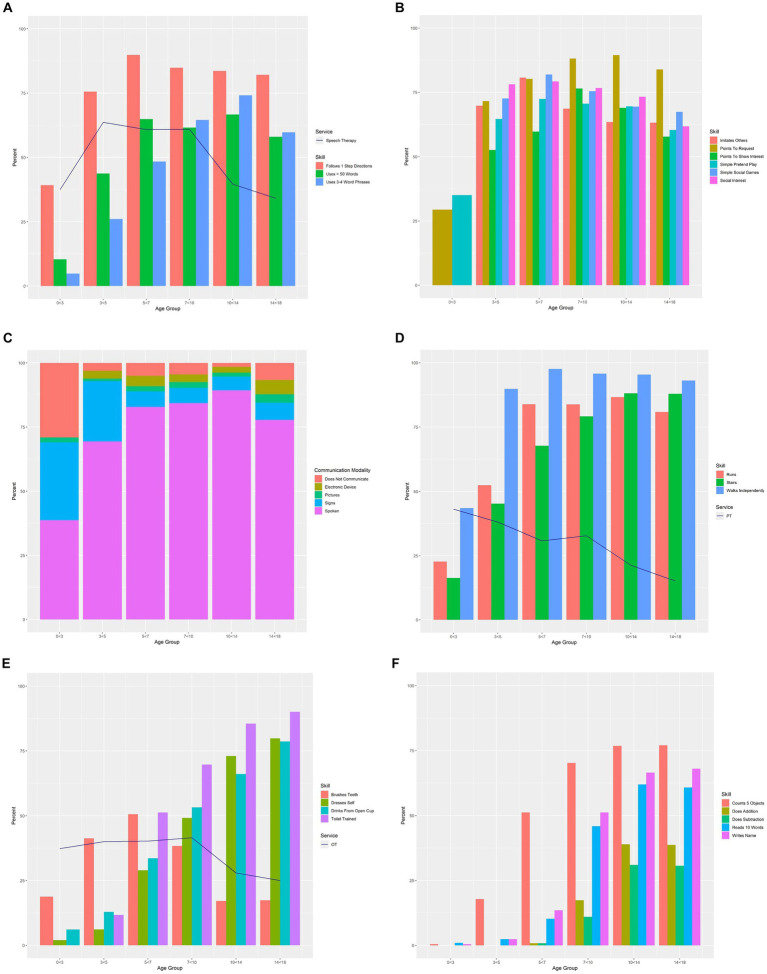
**(A)** Language skills by age group overlaid by receipt of speech therapy. Percentages of individuals having “established” select language skills per caregiver report on the General Development form. Individuals are represented only once per age group, with the most recent visit used when there were multiple visits in the same age-band. The percentage of individuals with established language skills in each age group is overlaid with receipt of speech therapy. **(B)** Social communication skills by age group. Percentages of individuals with “established” social communication skills per caregiver report on the Neurodevelopmental Parent Report for Outcome Monitoring (ND-PROM) and the General Development form. Individuals are represented only once per age group, with the most recent visit used when there were multiple visits in the same age-band. Social communication skills from the ND-PROM were not analyzed for 0 < 3-year-olds because the ND-PROM is only administered to patients ages 3 years and older. Additionally, “Points to Indicate Need for Help” from the General Development form was used as a proxy for “Points To Request” for the 0 < 3 years age group. **(C)** Communication modality by age group. Percentages of individuals using spoken language, sign language, pictures, or electronic communication devices, or not communicating at all, per caregiver report on the General Development form. Individuals are represented only once per age group, with the most recent visit used when there were multiple visits in the same age-band. **(D)** Gross motor skills by age group overlaid by receipt of physical therapy. Percentages of individuals having “established” select gross motor skills per caregiver report on the General Development form. Individuals are represented only once per age group, with the most recent visit used when there were multiple visits in the same age-band. The percentage of individuals with established gross motor skills in each age group is overlaid with receipt of physical therapy (PT = physical therapy). **(E)** Adaptive skills by age group overlaid by receipt of occupational therapy. Percentages of individuals with “established” adaptive skills per caregiver report on the Neurodevelopmental Parent Report for Outcome Monitoring (ND-PROM) and the General Development form. Individuals are represented only once per age group, with the most recent visit used when there were multiple visits in the same age-band. The percentage of individuals with established adaptive skills in each age group is overlaid with receipt of occupational therapy. Toilet Training was not analyzed for 0 < 3-year-olds because the ND-PROM is only administered to patients ages 3 years and older (OT = occupational therapy). **(F)** Academic skills by age group. Percentages of individuals having “established” select academic skills per caregiver report on the General Development form. Individuals are represented only once per age group, with the most recent visit used when there were multiple visits in the same age-band.

In order to provide an overview of developmental level of our clinical population, we examined attainment of skills (Language, Social Communication, Gross Motor, Adaptive, and Academic) and communication modality in each developmental domain, by age (Figures 1A–F).

#### 0 < 3 years

Expressive language skills were still emerging before age three years (Uses >50 Words/Signs/Phrases: 10%; Uses 3–4 Word Sentences: 5%), and very few used Picture Communication (2%). Many achieved Walking Independently (44%) and Following 1-Step Directions (39%), and about a third Pointed to Request (29%).

#### 3 < 5 years

By age five, about half of children Used >50 Words/Signs/Phrases (44%). Most communicated with Spoken Language (69%) and some used Sign Language (23%). Nearly two-thirds of three-to five-year-olds demonstrated Simple Pretend Play actions (65%) and more children were beginning to brush their teeth (“Brushes Teeth”; 41%). Almost all children were Walking Independently (90%) and over half were Running (52%).

#### 5 < 7 years

Nearly two-thirds of children Used >50 Words/Signs/Phrases (65%) by age seven years and about a half Used 3–4 Word Sentences (48%). Spoken Language was used most frequently (83%). More independent adaptive skills also began to emerge between ages five and seven years; for example, over a quarter dressed themselves (“Dresses Self”; 29%) and over half brushed their teeth (“Brushes Teeth”; 51%). Half were Toilet Trained (51%) and Counted At Least 5 Objects (51%).

#### 7 < 10 years

Between ages seven and 10 years, most children Used 3–4 Word Sentences (65%), relying on Spoken Language (84%) to communicate. A small number (4%) Did Not Communicate using any verbal or nonverbal communication modality. Adaptive skills continued to establish: some brushed their teeth (“Brushes Teeth”; 38%) and dressed themselves (“Dresses Self”; 49%), and most were Toilet Trained (70%). With regard to academic skills, many Counted At Least 5 Objects (70%), Read At Least 10 Words (46%), and wrote their name (“Writes Name”; 51%). Some Knew Basic Addition (17%) and Basic Subtraction (11%).

#### 10 < 14 years and 14 < 18 years

Expressive language skills and adaptive skills were well established by fourteen years of age in most children. Almost all used Spoken Language (89%), with fewer using alternative communication strategies. A very small number Did Not Communicate (2%). Academically, the majority of children Counted At Least 5 Objects (77%), Read At Least 10 Words (62%), and wrote their name (“Writes Name”; 51%) by age fourteen years. Some Knew Basic Addition (39%) and Basic Subtraction (31%). Between ages 14 and 18 years, similar patterns were noted.

### Behavior

**Figure 2:**
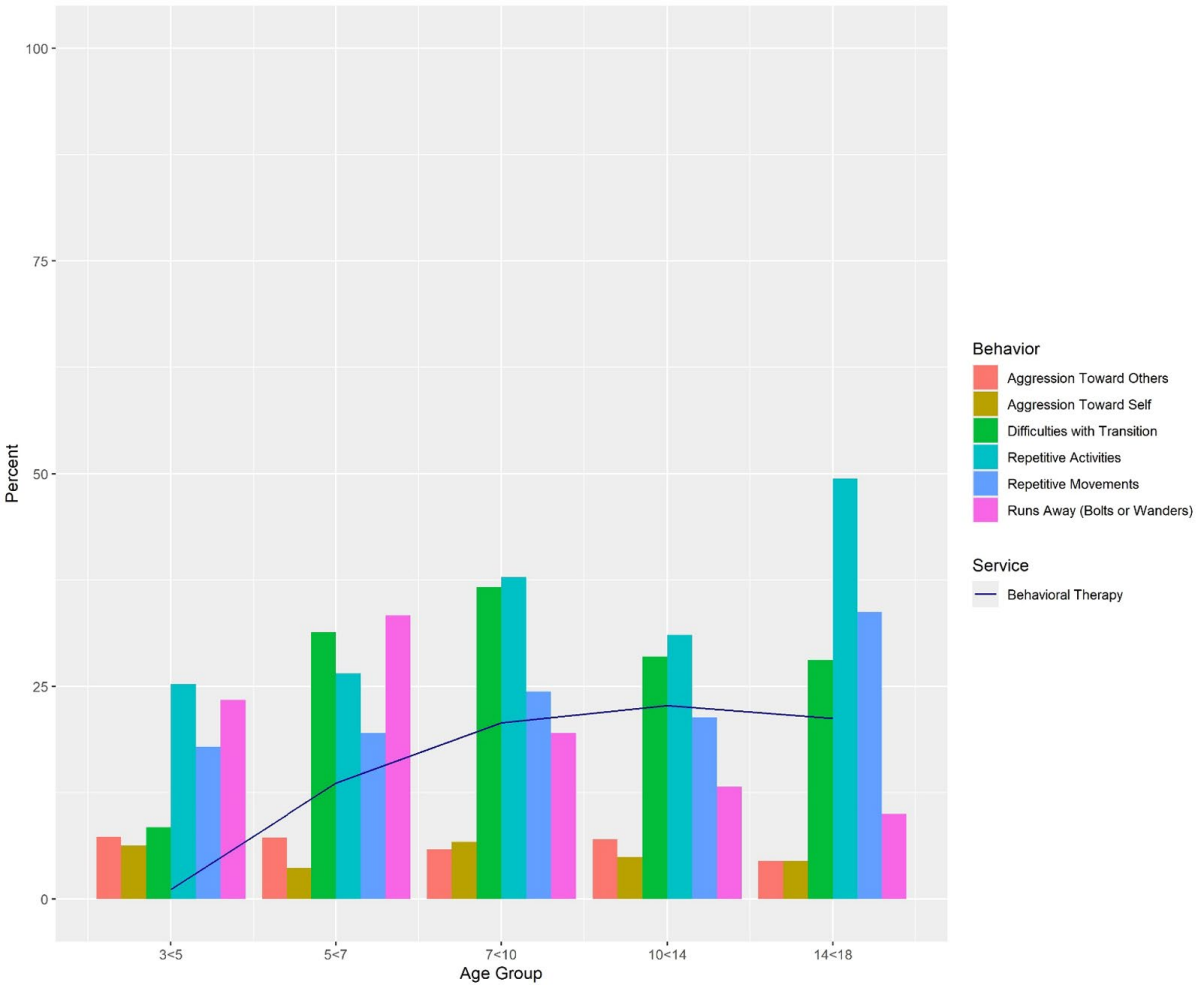
Behaviors by age group overlaid by receipt of behavioral therapy. Percentages of individuals with “established” maladaptive behaviors per caregiver report on the Neurodevelopmental Parent Report for Outcome Monitoring (ND-PROM). Individuals are represented only once per age group, with the most recent visit used when there were multiple visits in the same age-band. The percentage of individuals exhibiting behaviors in each age group is overlaid with receipt of behavioral therapy, which includes applied behavioral analysis (ABA). Behaviors were not analyzed for 0 < 3-year-olds because the ND-PROM is only administered to patients ages 3 years and older.

Behavioral challenges were listed as a Caregiver’s Chief Clinical Concern by 42% of caregivers. Additional behavior details and the achievement of milestones by age group are outlined below and in Figure 2. Behavioral challenges occurred in children ages three to five years, with a quarter reporting Repetitive Activities (25%) and Running Away (23%). Running Away was most common between ages five and seven years (33%), which is also when Difficulties with Transition became more frequent (31%). Nearly half of individuals in the oldest age group reported Repetitive Activities (49%). Aggression Toward Others and Aggression Toward Self were the least frequently reported behavioral challenges across all age groups.

### Education and developmental therapies

**Figure 3:**
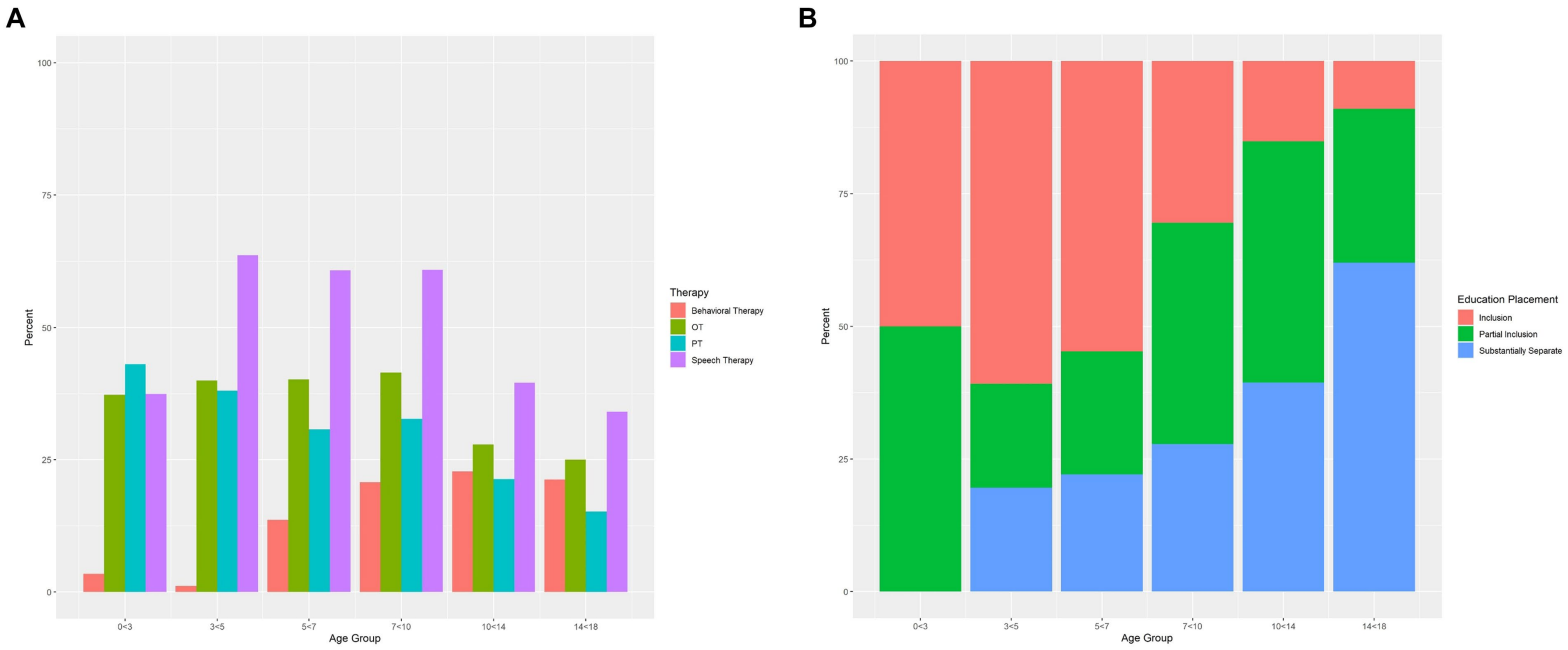
**(A)** Receipt of therapies by age group. Percentages of individuals receiving behavioral therapy, occupational therapy, physical therapy, and speech therapy per caregiver report on the Education and Services form. Individuals are represented only once per age group, with the most recent visit used when there were multiple visits in the same age-band. (OT = occupational therapy; PT = physical therapy). **(B)** Education placement by age group. Percentages of individuals in Inclusion (child is integrated into the general education classroom), Partial Inclusion (child is integrated into the general education classroom for some of the school day), or Substantially Separate (child is in a low teacher-to-student ratio setting rather than the general education classroom) educational settings per caregiver report on the Education and Services form. Individuals are represented only once per age group, with the most recent visit used when there were multiple visits in the same age-band.

We explored the rates of therapeutic service receipt and education placement by age group in our sample. Further information about specific therapies (Behavioral, Occupational, Physical, and Speech) and educational placement types (Inclusion, Partial Inclusion, and Substantially Separate) can be found in Figures 3A,B. Details about barriers to services (Waitlists, Insurance Coverage/Finances, Provider Availability, Transportation, Lack of Necessary Information About How to Access Services, and Program Not Willing/Able to Accommodate Child’s Needs) can be found in Table 3.

**Table 3:** Barriers to services by age group.

	Age Group (years)					
Barrier	0 < 3	3 < 5	5 < 7	7 < 10	10 < 14	14 < 18
Waitlists	35 (40%)	30 (53%)	31 (42%)	54 (46%)	61 (34%)	52 (44%)
Insurance coverage/finances	34 (24%)	24 (33%)	26 (15%)	55 (40%)	56 (34%)	44 (25%)
Provider availability	41 (42%)	26 (35%)	32 (41%)	56 (39%)	64 (45%)	54 (44%)
Transportation	33 (6%)	21 (10%)	26 (4%)	48 (13%)	58 (17%)	41 (10%)
Lack of necessary info about services	38 (32%)	29 (52%)	27 (30%)	54 (35%)	56 (45%)	47 (40%)
Program not willing/able to accommodate child’s needs	36 (25%)	23 (22%)	31 (26%)	53 (43%)	61 (25%)	49 (31%)
Other	39 (36%)	24 (46%)	30 (33%)	51 (20%)	63 (44%)	46 (35%)

Percentages of individuals experiencing barriers to services per caregiver report on the Education and Services form. Individuals are represented only once per age group, with the most recent visit used when there were multiple visits in the same age-band. “Other” responses included program closures/delays due to the COVID-19 pandemic; loss of program funding; lack of adequate staffing at programs; and not hearing back from programs after contacting them.

#### 0 < 3 years

The majority of children below three years of age (91%) received EI services, and many (53%) received Speech Therapy through EI. Some attended Daycare (23%). Very few children under three years of age received Behavioral Therapy through EI (3%). Almost no one reported receiving Behavioral Therapy outside of EI (1%). While many families received Services (87%), some encountered barriers, such as lack of Provider Availability (42%) and Necessary Information About How to Access Services (32%). Over half of children participated in Recreational Activities (55%).

#### 3 < 5 years

Most children of this group were in an Inclusion setting (61%), while fewer participated in Partial Inclusion (20%) or Substantially Separate (20%) classrooms (i.e., attending a small teacher-to-student ratio classroom specific for special education needs). Many children received services outside of their school system, including Speech and Language Therapy (64%), Physical Therapy (38%), and Occupational Therapy (40%). Hardly any children received Behavioral Therapy outside of school (1%). The majority of families received Services (80%), while some encountered barriers (19%). Over three quarters of children participated in Recreational Activities (78%).

#### 5 < 7 years

In this age group, some were taught in an Inclusion classroom (55%), while others attended a Partial Inclusion (23%), and/or Substantially Separate classroom (22%). Services were commonly received outside of school (Speech and Language Therapy: 61%; Physical Therapy: 31%; Occupational Therapy: 40%). Some five-to seven-year-olds also accessed Behavioral Therapy outside of school (14%). Most families received Services (78%), while some encountered barriers (12%). The majority of children participated in Recreational Activities (78%).

#### 7 < 10 years

Between the ages of seven and ten, many (42%) were in a Partial Inclusion classroom setting, with fewer in an Inclusion classroom (30%) or Substantially Separate classroom (28%). Non-school services were frequently received (Speech and Language Therapy: 61%; Physical Therapy: 33%; Occupational Therapy: 42%). Behavioral Therapy was received by some outside of school (21%). Most families received Services (74%), and many encountered barriers to services (18%). The vast majority of children were involved in Recreational Activities (81%).

#### 10 < 14 years

Only some of ten-to fourteen-year-olds were in an Inclusion classroom (15%), with most placed in either a Partial Inclusion classroom (45%) or Substantially-Separate classroom (39%). Many received Speech and Language Therapy (40%) and others accessed Physical (21%) and Occupational Therapy (28%) outside of school. Some received Behavioral Therapy outside of school (23%). About a third (32%) faced barriers. Almost all were involved in Recreational Activities (85%).

#### 14 < 18 years

In this age group, most children were placed in a Substantially-Separate classroom (62%), while fewer remained in a Partial Inclusion (29%) or Inclusion classroom (9%). Some received non-school Speech and Language Therapy (34%) and a quarter received private Occupational Therapy (25%). Only a few received Physical Therapy (15%) and Behavioral Therapy (21%) outside of school. It was common for families to face barriers (31%). Almost all were involved in Recreational Activities (90%).

## Discussion

We describe general developmental skills, behavior, and receipt of education and services for a large cohort of children, adolescents, and young adults with DS, who are clinically followed in a specialized DSP in the United States. Here, we provide a snapshot of developmental skills by age that may be anticipated by clinicians based on a large number of patients with DS. General developmental trends are consistent with prior reports (Dykens et al., 2006; Onnivello et al., 2023; Buckley and Bird, 1993; Baumer et al., 2024; Sella et al., 2021; Laws et al., 2000; Turner et al., 2008; Centers for Disease Control and Prevention, 2023). While delays were present across all developmental domains, those related to language skills, academic skills, and toileting skills were most substantially delayed relative to established milestones for children without DS (Zubler et al., 2022). For example, only about half of our sample were using 50 words, pictures, or signs by age five, whereas in a typical population this skill attainment is anticipated between two and three years (Zubler et al., 2022). Academically, literacy and math skills would typically be established by age five to six in children without DS (The Common Core. English Language Arts Standards, 2021; The Common Core. Mathematics Standards, 2021); in our sample we found that by age 10 years only about a third of individuals had established basic addition and subtraction while about two-thirds had established early literacy skills. Toileting is also typically achieved by children between 24 and 48 months (Clifford and Gorodzinsky, 2000); however, the majority of our sample were not toilet trained until much later.

In contrast, early play and social communication skills were more closely aligned with age-expected developmental achievement. For example, more than half of the three-to five-year olds in our sample demonstrated simple pretend play skills; in a typically-developing population, 75% of children are estimated to have this skill at age four (Zubler et al., 2022).

To the best of our knowledge, this is one of the first reports of current service receipt in a US-based clinical DS sample. While this data was reported in a database in a purportedly high-resourced area (Friedman-Krauss and Barnett, 2023), and in a clinical population in which families are provided expert guidance about recommended services, there were many potential gaps identified. Almost all children received EI services, and many young children aged three to ten received speech, occupational, and physical therapies outside of school. Older participants demonstrated more impairment (e.g., more communication challenges, an increase in some behavioral challenges, etc.) than younger participants, perhaps indicative of our clinic sample and the need for continued specialty care. Still, the greatest potential gaps were noted in older children; while younger children accessed services at a high rate, older children did not frequently receive services such as speech therapy and occupational therapy, despite ongoing developmental and behavioral challenges. This was particularly true for non-public services, which decreased for school-aged children as they grew older. Barriers to services were reported in up to one-third of the population, including waitlists/lack of providers, inadequate information about services and how to access them, and service delivery changes due to COVID-19. Regardless of skill acquisition, caregivers shared developmental concerns and worries about behavioral challenges across age groups. Recreational activities were well-represented across age groups. Additional notably important themes are described here by domain.

### Speech and language services

Ongoing challenges with verbal communication were evident in the study population. Most children younger than seven were not yet speaking in short sentences. Young children in clinic are provided with routine counseling on the benefits of using manual signs to increase early communication skills (Clibbens, 2001), yet very few individuals used alternative communications. Further, despite the trends in delayed communication skills, there was minimal accessing of Speech Therapy and language-based intervention by children across ages in our sample. This suggests a greater need for speech and language therapy and the use of assistive technology and functional strategies to maximize communication especially as spoken language skills are developing.

### Motor and adaptive skills

Very few children were walking between ages zero and three, and about half received physical therapy privately or through EI. Adaptive skills began emerging in school-age children. Only half of children achieved toilet training by age seven years, but few received additional behavioral therapies, which could be used to target toileting needs. Motor and adaptive skills were relatively well-established by 14 years of age, which is reflected by fewer individuals (approximately one-fourth) receiving occupational and physical therapies.

### Behavioral therapies and supports

Caregivers reported behavioral challenges to clinicians as a primary concern in about half of individuals. Behavioral challenges were high across age groups and have substantial consequences for educational, social, and family functioning. Despite these frequent concerns regarding behavior, behavioral therapies were infrequently accessed. Given the known benefit of behavioral interventions for promoting developmental skills and reducing challenging behaviors in children with developmental disabilities (Feeley and Jones, 2008), this is an area of great need for children with DS. Children with other neurodevelopmental disabilities, such as ASD, are able to access behavioral services much more easily than children with DS (Kaat and Lecavalier, 2013). However, individuals with DS display many similar behavioral challenges, including repetitive behavior, and some deficits in social communication skills, for which behavioral interventions may be effective (Ivy and Schreck, 2016). Given the prevalence of unsafe behaviors, such as running away and aggression toward self and others, across age groups, safety counseling and behavior management teaching and intervention should be available for individuals with DS at all ages. Additionally, as skill deficits may contribute to behavioral challenges, comprehensive services are needed to address these common concerns.

### Education and services

Academic achievement continues to develop into adolescence. As such, tailored instructional methods are needed to help individuals with DS make effective progress. Numeracy skills are essential for financial independence, health, and adaptive functioning (Sella et al., 2021). Research has shown that children with DS have strong visual-matching abilities and are capable of learning sight words at a very young age (between 2.5 and 3.5 years) (Buckley and Bird, 1993); however, very few children under the age of five in our sample have reported pre-literacy or early reading skills. Though we did not statistically compare skills and services, we were able to examine concurrent skills and receipt of services within our sample and describe general trends. While research-informed services exist, including phonics-based instructional methods, which tap into reported strengths of children with DS (Lemons and Fuchs, 2010), it is unclear whether children in our sample were receiving this type of instruction. More research is needed to understand how instructional practices can be utilized to maximize academic potential in children with DS. With regards to educational placement, rates of placement in inclusion educational settings were relatively high in preschool and early elementary school years, but they dropped dramatically in middle and high school. Given prior research that inclusion for individuals with DS results in more developed language and social skills, as well as higher academic achievement (Laws et al., 2000; Turner et al., 2008), advocacy should promote inclusive educational programming.

In summary, our clinical database of children, adolescents, and young adults is one of the largest clinical DS samples that currently exists, and enables us to examine the current state of developmental skills and service receipt in this population. The findings of this study can serve to further inform clinical care and service delivery policy, and have implications for future public health and educational initiatives in DS.

#### Limitations

The study serves as a snapshot of development and services received by a large number of patients in a relatively high resourced area; thus, our cohort is not representative of the broader population of the United States and the generalizability of the results is limited. As such, the results of this study may reflect developmental changes in those with DS who receive more support through EI and special needs education compared to other populations. Additionally, given that the individuals in this study are followed in the clinic until they transition to adult services, it is possible that many choose to continue to receive care in the clinic because they have complex needs, which may influence development, behavior, and services reported. Secondly, though the database involved standardizing clinical practice across several providers of different training backgrounds, it did not exclusively involve the use of standardized data collection tools. Given the approach of collecting information in all clinic visits, there are variations in the number of individuals who completed each of the measures. Third, caregiver report of services may be misrepresented in some areas. For example, caregivers may have misread or misunderstood sections of forms and noted non-public services instead of EI or school programming services. Fourth, because we reviewed collected data on milestones at the time of each clinical visit, the exact timing of milestone achievement was not available. Additionally, the data were collected prior, during, and after the COVID-19 pandemic, but did not specifically look at types of services that continued or discontinued during the COVID-19 timeframe. Finally, the expertise of the clinicians in areas like toileting and educational supports may draw families who are in greater need of those services.

#### Future directions

Future studies should investigate correlations between development and services in this population, and should extend across all ages, including adults. Further investigation into the potential confounding impacts and relationships of certain developmental domains (e.g., the attainment of gross motor milestones) on cognitive and academic skills must be explored (Will et al., 2018). It is also important to consider the impact of social isolation and the interruption of services during the COVID-19 pandemic period.

## Conclusion

Systematic collection of clinical data in a large cohort of individuals with DS can guide and enhance specialized care for DS. In this cohort, many individuals had high levels of need but did not receive commensurate services or support. Despite the prevalence of delays and behavioral challenges across domains, and the presence of language delays and behavioral challenges, therapies to support these levels of need were reported at much lower frequencies. There is important need for improving access to tailored interventions. These findings may help to inform policy change related to developmental and educational services for individuals with DS.

## Data Availability

The original contributions presented in the study are included in the article/supplementary material, further inquiries can be directed to the corresponding author.

## References

[ref1] Barger M. M. Kim E. M. Kuncel N. R. Pomerantz E. M. (2019). The relation between parents’ involvement in children’s schooling and children’s adjustment: a meta-analysis. Psychol. Bull. 145, 855–890. doi: 10.1037/bul0000201, PMID: 31305088

[ref2] Baumer N. T. Hojlo M. A. Lombardo A. M. Milliken A. L. Pawlowski K. G. Sargado S.. (2022). Development and implementation of a longitudinal clinical database for down syndrome in a large pediatric specialty clinic: methodology and feasibility. J. Intellect. Disabil. 28, 196–215. doi: 10.1177/17446295221133874 36245216

[ref3] Baumer N. T. Pawlowski K. Zhang B. Sideridis G. (2024). Validation of factor structure of the neurodevelopmental parent report for outcome monitoring (ND-PROM) in down syndrome: confirmatory factor analysis. Front. Psychiatry 15:1293937. doi: 10.3389/fpsyt.2024.1293937 38505792 PMC10948425

[ref4] Buckley S. Bird G. (1993). Teaching children with down syndrome to read. Downs Syndr. Res. Pract. 1, 34–39. doi: 10.3104/perspectives.9

[ref5] Centers for Disease Control and Prevention. Facts about Down Syndrome. Centers for Disease Control and Prevention. (2023). Available at: https://www.cdc.gov/ncbddd/birthdefects/downsyndrome.html (accessed Sep 22, 2023).

[ref6] Chapman R. S. Hesketh L. J. (2001). Language, cognition, and short-term memory in individuals with down syndrome. Downs Syndr. Res. Pract. 7, 1–7. doi: 10.3104/reviews.108 11706807

[ref7] Clibbens J. (2001). Signing and lexical development in children with down syndrome. Downs Syndr. Res. Pract. 7, 101–105. doi: 10.3104/reviews.119, PMID: 11721535

[ref8] Clifford T. Gorodzinsky F. (2000). Toilet learning: anticipatory guidance with a child-oriented approach. Paediatr. Child Health 5, 333–335. doi: 10.1093/pch/5.6.333, PMID: 20177551 PMC2819951

[ref9] de Graaf G. Van Hove G. Haveman M.. Effects of regular versus special school placement on students with down syndrome: a systematic review of studies. In: Dubois A. Bosch E. van den, editors. New developments in down syndrome research. Hauppauge, NY, USA: Nova Science Publishers Inc.; (2012). p. 45–86.

[ref10] Dykens E. Hodapp R. Evans D. (2006). Profiles and development of adaptive behavior in children with down syndrome. Downs Syndr. Res. Pract. 9, 45–50. doi: 10.3104/reprints.293, PMID: 16869374

[ref11] Feeley K. Jones E. (2008). Strategies to address challenging behaviour in young children with down syndrome. Downs Syndr. Res. Pract. 12, 153–163. doi: 10.3104/case-studies.2008, PMID: 19026289

[ref12] Frank K. Esbensen A. J. (2015). Fine motor and self-care milestones for individuals with down syndrome using a retrospective chart review. J. Intellect. Disabil. Res. 59, 719–729. doi: 10.1111/jir.12176, PMID: 25533735

[ref13] Friedman-Krauss A. H. Barnett W. S. (2023). The state(s) of early intervention and early childhood special education: looking at equity. New Brunswick, NJ: National Institute for Early Education Research. Available at: https://nieer.org/research-library/states-early-intervention-early-childhood-special-education

[ref14] Hargreaves S. Holton S. Baxter R. Burgoyne K. (2021). Educational experiences of pupils with down syndrome in the UK. Res. Dev. Disabil. 119:104115. doi: 10.1016/j.ridd.2021.104115 34736106

[ref15] Harris P. A. Taylor R. Minor B. L. Elliott V. Fernandez M. O’Neal L.. (2019). The REDCap consortium: building an international community of software platform partners. J. Biomed. Inform. 95:103208. doi: 10.1016/j.jbi.2019.103208, PMID: 31078660 PMC7254481

[ref16] Harris P. A. Taylor R. Thielke R. Payne J. Gonzalez N. Conde J. G. (2009). Research electronic data capture (REDCap)—a metadata-driven methodology and workflow process for providing translational research informatics support. J. Biomed. Inform. 42, 377–381. doi: 10.1016/j.jbi.2008.08.010, PMID: 18929686 PMC2700030

[ref17] Hendrix J. A. Amon A. Abbeduto L. Agiovlasitis S. Alsaied T. Anderson H. A.. (2021). Opportunities, barriers, and recommendations in down syndrome research. Transl. Sci. Rare Dis. 5, 99–129. doi: 10.3233/trd-200090, PMID: 34268067 PMC8279178

[ref18] Individuals with Disabilities Education Act. USA: Subchapter 1 (Part A), (1975). Available at: https://sites.ed.gov/idea/statute-chapter-33/subchapter-i

[ref19] Ivy J. W. Schreck K. A. (2016). The efficacy of ABA for individuals with autism across the lifespan. Curr. Dev. Disord. Rep. 3, 57–66. doi: 10.1007/s40474-016-0070-1

[ref20] Kaat A. J. Lecavalier L. (2013). Disruptive behavior disorders in children and adolescents with autism spectrum disorders: a review of the prevalence, presentation, and treatment. Res. Autism Spectr. Disord. 7, 1579–1594. doi: 10.1016/j.rasd.2013.08.012

[ref21] Laws G. Byrne A. Buckley S. (2000). Language and memory development in children with down syndrome at mainstream schools and special schools: a comparison. Educ. Psychol. 20, 447–457. doi: 10.1080/713663758

[ref22] Lemons C. J. Fuchs D. (2010). Phonological awareness of children with down syndrome: its role in learning to read and the effectiveness of related interventions. Res. Dev. Disabil. 31, 316–330. doi: 10.1016/j.ridd.2009.11.002, PMID: 19945821

[ref23] Levin A. R. Baumer N. Amaral J. Sargado S. Pawlowski K. Chiujdea M.. (2021). Autism Spectrum disorder parent report for outcome monitoring: a preliminary report of development and clinical utility. J. Dev. Behav. Pediatr. 42, 272–282. doi: 10.1097/DBP.0000000000000895, PMID: 33394835

[ref24] Luyster R. J. Seery A. Talbott M. R. Tager-Flusberg H. (2011). Identifying early-risk markers and developmental trajectories for language impairment in neurodevelopmental disorders. Dev. Disabil. Res. Rev. 17, 151–159. doi: 10.1002/ddrr.1109 23362034

[ref25] Martin G. E. Klusek J. Estigarribia B. Roberts J. E. (2009). Language characteristics of individuals with down syndrome. Top. Lang. Disord. 29, 112–132. doi: 10.1097/TLD.0b013e3181a71fe1, PMID: 20428477 PMC2860304

[ref26] Onnivello S. Schworer E. K. Daunhauer L. A. Fidler D. J. (2023). Acquisition of cognitive and communication milestones in infants with down syndrome. J. Intellect. Disabil. Res. 67, 239–253. doi: 10.1111/jir.12893 34761472

[ref27] R Core Team (2024). R: A language and environment for statistical computing. Vienna, Austria: R Foundation for Statistical Computing.

[ref28] Roizen N. J. Patterson D. (2003). Down’s syndrome. Lancet 361, 1281–1289. doi: 10.1016/S0140-6736(03)12987-X 12699967

[ref29] Sella F. Onnivello S. Lunardon M. Lanfranchi S. Zorzi M. (2021). Training basic numerical skills in children with down syndrome using the computerized game “the number race”. Sci. Rep. 11:2087. doi: 10.1038/s41598-020-78801-5, PMID: 33483541 PMC7822821

[ref30] Sigman M. Ruskin E. (1999). Chapter I: background and goals of this study. Monogr. Soc. Res. Child Dev. 64, 1–10. doi: 10.1111/1540-5834.00002 10412222

[ref31] The Common Core. English Language Arts Standards. Common Core State Standards Initiative. (2021). Available at: https://www.thecorestandards.org/ELA-Literacy/

[ref32] The Common Core. Mathematics Standards. Common Core State Standards Initiative. (2021). Available at: https://www.thecorestandards.org/Math/

[ref33] Turner S. Alborz A. Gayle V. (2008). Predictors of academic attainments of young people with Down’s syndrome. J. Intellect. Disabil. Res. 52, 380–392. doi: 10.1111/j.1365-2788.2007.01038.x, PMID: 18205756

[ref34] Vellody K. (2020). Developmental expectations and medical issues for children with down syndrome. Roswell, GA, USA: National Down Syndrome Congress. Available at: https://www.ndsccenter.org/wp-content/uploads/MilestonesMed-Issues.pdf

[ref35] Will E. A. Caravella K. E. Hahn L. J. Fidler D. J. Roberts J. E. (2018). Adaptive behavior in infants and toddlers with down syndrome and fragile X syndrome. Am. J. Med. Genet. B Neuropsychiatr. Genet. 177, 358–368. doi: 10.1002/ajmg.b.32619, PMID: 29399949 PMC7294771

[ref36] Zubler J. M. Wiggins L. D. Macias M. M. Whitaker T. M. Shaw J. S. Squires J. K.. (2022). Evidence-informed milestones for developmental surveillance tools. Pediatrics 149:e2021052138. doi: 10.1542/peds.2021-052138, PMID: 35132439 PMC9680195

